# Physical Activity, Mind Wandering, Affect, and Sleep: An Ecological Momentary Assessment

**DOI:** 10.2196/mhealth.5855

**Published:** 2016-08-31

**Authors:** Jason Fanning, Michael Mackenzie, Sarah Roberts, Ines Crato, Diane Ehlers, Edward McAuley

**Affiliations:** ^1^ Exercise Psychology Lab Department of Kinesiology and Community Health University of Illinois at Urbana-Champaign Urbana, IL United States; ^2^ Mind Body Behavior Laboratory Department of Behavioral Health & Nutrition University of Delaware Newark, DE United States

**Keywords:** physical activity, mHealth, attention, sleep, affect

## Abstract

**Background:**

A considerable portion of daily thought is spent in mind wandering. This behavior has been related to positive (eg, future planning, problem solving) and negative (eg, unhappiness, impaired cognitive performance) outcomes.

**Objective:**

Based on previous research suggesting future-oriented (ie, prospective) mind wandering may support autobiographical planning and self-regulation, this study examined associations between hourly mind wandering and moderate-to-vigorous physical activity (MVPA), and the impact of affect and daily sleep on these relations.

**Methods:**

College-aged adults (N=33) participated in a mobile phone-delivered ecological momentary assessment study for 1 week. Sixteen hourly prompts assessing mind wandering and affect were delivered daily via participants’ mobile phones. Perceived sleep quality and duration was assessed during the first prompt each day, and participants wore an ActiGraph accelerometer during waking hours throughout the study week.

**Results:**

Study findings suggest present-moment mind wandering was positively associated with future MVPA (*P*=.03), and this relationship was moderated by affective state (*P*=.04). Moreover, excessive sleep the previous evening was related to less MVPA across the following day (*P*=.007). Further, mind wandering was positively related to activity only among those who did not oversleep (*P*=.007).

**Conclusions:**

Together, these results have implications for multiple health behavior interventions targeting physical activity, affect, and sleep. Researchers may also build on this work by studying these relationships in the context of other important behaviors and psychosocial factors (eg, tobacco use, depression, loneliness).

## Introduction

Approximately half of waking thought is unrelated to events occurring in the moment [1]. Despite being a predominant form of thought, researchers are only beginning to understand the role of task-independent thought (ie, mind wandering) in daily functioning. Results from numerous studies suggest thought unrelated to the task at hand is often prospective in nature (ie, focused on the future), which may play an important role in regulating and managing future behavior [2,3]. Moreover, it has been suggested that our ability for future-oriented mind wandering supports patience over impulsivity [4], and may be crucial for creative thought and problem solving [5,6].

Despite the apparent value of task-independent thought, some have cautioned that a number of factors related to the individual or to the tasks in which they are engaged may modulate the nature of the effect of mind wandering on daily functioning and health [6]. For instance, Baird and colleagues [2] noted that during times of low affect, high stress, or during complex tasks, task-independent thoughts tended to shift toward past experiences. Indeed, a number of studies have found this type of retrospective mind wandering is often ruminative in nature and associated with a variety of deleterious outcomes ranging from impaired cognitive performance (eg, reduced working memory function, reading comprehension) [7-10] to accelerated cellular aging [11]. Many of these negative findings have been noted in studies conducted in and out of the research laboratory. For instance, in a seminal study, Killingsworth and Gilbert [1] utilized mobile phone-based ecological momentary assessment (EMA) techniques [12] to examine the relationship between mind wandering and affect throughout the day in a sample of 2250 adults. The researchers reported individuals were less happy during periods when their minds wandered, and this occurred regardless of the activity in which the individual was currently engaged. Other investigators have drawn similar connections, reporting associations between negative affect and increased mind wandering during reading, and an increased likelihood for retrospective thought [13,14]. Moreover, mind wandering appears to be both a cause and consequence of negative affect [13-15].

Because affective valence and other statelike psychological factors appear to influence the tendency for one’s wandering thoughts to be prospective or retrospective in nature, mind wandering may be of particular importance in the daily self-regulation of a number of complex health behaviors, including physical activity. Regular participation in moderate-to-vigorous physical activity (MVPA) is vital to health across the life span, and failure to engage in sufficient levels of the behavior has been related to increased risk for myriad disease states, including cardiovascular disease, type 2 diabetes, several types of cancer, and osteoporosis among others [16]. Still, only half of adults in the United States participate in levels of physical activity sufficient to achieve health benefits [17]. This may be due, in part, to its nature as a challenging and complex behavior that requires considerable self-regulatory skill to maintain, particularly in the face of modern technologies and environments that often incentivize inactivity [18,19]. Although limited research has examined factors impacting an individual’s activity behavior at the daily or hourly level, it may be expected that the nature of one’s mind wandering may impact one’s ability to plan for, and engage in, this important behavior.

A number of lifestyle factors likely play an important role in the ongoing relationship between mind wandering and physical activity, and one’s affective state and sleep habits may be expected to be particularly important. As described previously, affect has a demonstrable impact on the nature of mind wandering, and one’s sleep habits are closely tied with both physical activity and affect. Indeed, poor sleep habits have been associated with a number of correlates of retrospective mind wandering, such as impaired working memory function [20,21] and negative affect [22-24]. Moreover, several studies have demonstrated that these habits contribute to lower levels of physical activity [25-27]. If one’s mind wandering is indeed associated with their participation in physical activity, sleep may be an important moderator of this relationship. More specifically, healthy sleep may predispose an individual to prospective mind wandering, in turn supporting participation in physical activity.

Researchers have not yet examined the relationship between mind wandering and MVPA, or the influence of potentially potent correlates such as affect and sleep. Thus, the purpose of this exploratory study is to extend previous research related to mind wandering by examining these relations at the hourly and daily level in a sample of college students. Because previous research suggests mind wandering is most often biased toward the future [13], we hypothesized that hourly mind wandering would indeed facilitate higher levels of accelerometer-measured MVPA in subsequent hours. Next, because affect has been demonstrated to modulate the tendency for mind wandering to focus on the future rather than past [2], we hypothesized that a positive affective state would also be associated with increased participation in subsequent MVPA. Importantly, we also hypothesized that this state would moderate the relationship between mind wandering and physical activity whereby during hours of positive affect, mind wandering would be related to increased physical activity. Conversely, during times of low affect, mind wandering would be related to less activity. Finally, given the prevalence of sleep disturbances in this population [28], and the documented associations between sleep, affect, and cognitive functioning, we hypothesized that better sleep (assessed via daily self-reported sleep quality and duration) would be related to greater MVPA.

## Methods

### Participants

A convenience sample of adults aged 18 to 25 years (N=36) were recruited via posted flyers and Web-based advertisement from a large Midwestern university to participate in a study investigating the relationships between mind wandering, affect regulation, and MVPA. Eligible participants were adults 18 to 25 years of age, able to communicate in English, able to walk independently, and owners of an Android or iPhone mobile phone with access to text messaging and mobile Internet.

### Procedures

#### The Ecological Momentary Assessment System

Mobile phone-delivered EMAs have increased in popularity in recent years, and for good reason. The devices are nearly ubiquitous (85% of young adults in the United States owned a mobile phone in 2014 [29]), and are carried with the individual at all times. Accordingly, researchers are able to collect and screen self-reported information from individuals as the behavior occurs and in the context in which it occurs [12,30]. This offers a unique opportunity to behavioral researchers, allowing for the examination of important psychological constructs and their interactions with an individual’s behavior at the daily or hourly level. Additionally, because data may be collected as a behavior occurs, EMA methods have the potential to decrease recall bias [12].

Importantly, recent advances in mobile phone and mobile Internet technologies have allowed for the development of EMA apps that are cross-platform compatible (ie, able to be used regardless of mobile operating system), automated, highly individualized, and easily scalable. The app used in this study was developed by one member of the research team (JF) using Perl, hypertext markup language, cascading style sheets, and JavaScript, and was integrated with the commercial text messaging service Twilio (Twilio, San Francisco, CA, USA) to provide automated text message prompts. These prompts were delivered at 10 minutes to the hour between 6:50 am and 9:50 pm to accommodate student class schedules. Each prompt contained a link to a survey tailored to the individual and the time of day (eg, sleep questions were delivered during the individual’s first answered survey of the day). Each survey took between 30 seconds and 1 minute to complete, and assessed a number of aspects of attention, including mind wandering, affect, and sleep. The Institutional Review Board of the University of Illinois approved all study procedures.

#### Study Progression

Interested individuals contacted the research staff via telephone and were screened for eligibility. Those who were eligible were provided an institutional review board-approved informed consent and completed a series of online questionnaires and demographic items. Following questionnaire completion, participants attended a brief orientation session. During this time, trained research staff introduced each participant to the mobile phone EMA system, and participants were familiarized with each question presented during the daily mobile phone-based surveys. Following this orientation appointment, participants began receiving prompts via text message to complete study assessments, and each participant wore an ActiGraph accelerometer during waking hours on each day of the study period. For this early study, a 7-day study period was selected to align with common accelerometer protocols concerned with capturing adults’ usual physical activity [31].

### Measures

#### Demographics

Demographic information was collected via telephone. Items included gender, race, ethnicity, and date of birth (used to calculate age at the time of screening).

#### Mind Wandering

**Figure 1:**
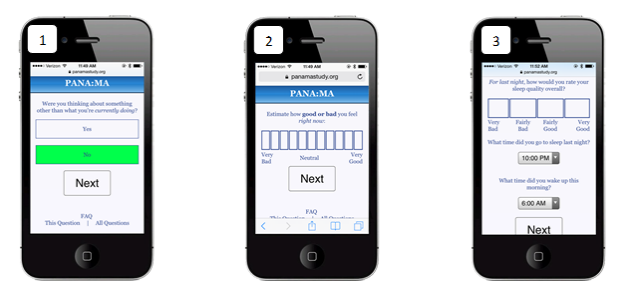
Panel 1 demonstrates the survey item assessing mind wandering; panel 2 demonstrates the survey item assessing affect; and panel 3 demonstrates the survey item assessing sleep.

Mind wandering was assessed each hour using an item adapted from Killingsworth and Gilbert [1]. This item asked, “Were you thinking about something other than what you were currently doing?” Response options were “yes” and “no” (see Figure 1, panel 1).

#### Affect

Affect was assessed each hour using the Feeling Scale [32]. This item asked the participant to “estimate how good or bad you feel right now.” Answers were provided on an 11-point Likert-type format with possible response options ranging from “very bad” to “very good” (see Figure 1, panel 2).

#### Sleep

Two sleep-related measures were adapted from the Pittsburgh Sleep Quality Index [33] and were administered during the first assessment of each day. To assess the previous night’s sleep quality, participants responded to the following statement: “For *last night*, how would you rate your sleep quality overall?” Response options were “very bad,” “fairly bad,” “fairly good,” and “very good.” Next, participants entered the previous night’s sleep and wake times, and these were used to calculate time spent sleeping (see Figure 1, panel 3).

#### Physical Activity

ActiGraph accelerometers (model GT3x+; ActiGraph, Pensacola, FL, USA) were used to objectively assess physical activity. Participants wore the device on the nondominant hip during waking hours for the duration of the study period. Data were downloaded as activity counts, reflecting raw accelerations summed over a specific epoch length (eg, 60 seconds [34]). These data were processed in ActiLife version 6.13.2 (ActiGraph, Pensacola, FL, USA) using cut points for adults [35]. The cut point denoting MVPA was ≥1952 counts per minute. Data were summed within 1-hour periods, and time-matched with hourly survey data. These data represented activity occurring within the hour in which the survey was completed. Finally, a time-lagged activity variable was created to represent physical activity occurring 1 hour beyond the completion of a given prompt.

### Data Analyses

First, the proportion of surveys completed during each hour between 6:50 am and 9:50 pm, and the proportion of all hourly surveys completed by each participant, were examined for missing data. Hours with a response rate less than 0.5 standard deviations of the mean were removed from the dataset. Due to the high number of potential prompts delivered per day, an a priori criterion of 25% completion per participant was employed. Such an approach is common to EMA protocols [36] **.** Next, skewness and kurtosis were examined to verify normality. Due to the nested structure of the data, such that observations were nested within persons, multilevel linear regression was used to conduct analyses [37]. Analyses followed a forward-stepping hierarchical approach in which fixed and random effects of linear and quadratic time, as well as day of week on hourly MVPA were tested. Next, fixed effects of person-level, average daily, and hourly mind wandering were examined. Hourly affect, nightly sleep quality and duration, and demographic factors were then tested. Finally, simple slopes analyses were conducted to aid in interpretation of significant interaction effects [38]. Model fit was assessed with –2 restricted log-likelihood, Akaike information criterion (AIC), and the Schwarz Bayesian information criterion (BIC). Predictors were retained in the model at *P*<.10, and were considered significant at *P*<.05. Demographics were described using descriptive statistics, and all data analyses were conducted in SPSS version 23 (IBM Corp, Armonk, NY, USA).

## Results

Participant characteristics are displayed in Table 1. The mean age of participants was 20.5 (SD 1.5) years, 75% (27/36) were female, and 50% (18/36) were white. Participants reported mind wandering on 41.11% (908/2204) of answered prompts. Preliminary analyses revealed that response rates to prompts delivered at 6:50 am and 7:50 am fell outside of the 0.5 SD range, and these observations were subsequently removed from the dataset. Additionally, three of 36 participants (8%) answered fewer than 25% (24/98) of the prompts and were removed from analyses. As a result, a total of 3234 person*hour*day observations across 33 participants were included in the analyses. On average, participants responded to 64% (63/98) of their prompts (range 27%-92%).

**Table 1:** Participant characteristics (N=36).

Measure		Participants
Age (years), mean (SD)		20.5 (1.5)
**Sex, n (%)**		
	Female	27 (75)
	Male	9 (25)
**Race, n (%)**		
	African American	3 (8)
	Asian	11 (31)
	Native American	2 (6)
	White	18 (50)
	Unknown/Other	2 (5)

**Figure 2:**
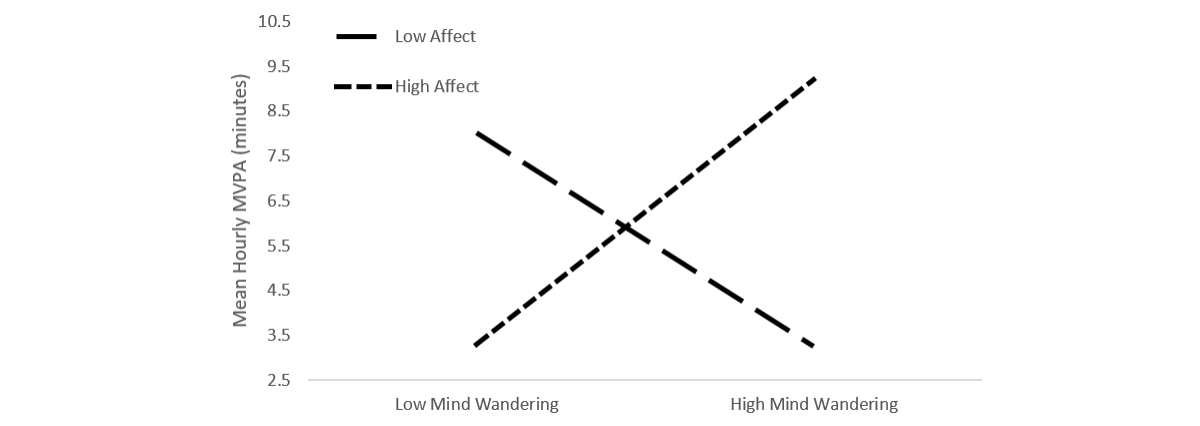
Interaction between mind wandering and affect.

As expected, results from the unconditional means model suggested a large amount of total variance in MVPA was within-person (intraclass correlation coefficient [ICC]=.05). Analyses revealed a significant negative linear (*P*=.001) and quadratic (*P*<.001) time effect for MVPA over the study period, and there was a significant positive relationship for day of week (*P*=.01) such that individuals tended to engage in greater MVPA as the week progressed. The degree to which an individual experienced mind wandering within an hour was not associated with MVPA during the hour (*P*=.47), but was positively associated with their participation in MVPA in the coming hour (*P*=.03). Hourly affect was also significantly and positively related to MVPA in the coming hour (*P*=.02), and there was a significant interaction between hourly affect and hourly mind wandering (*P*=.04; see model 1). Simple slopes analyses indicated that during times of greater positive affect, mind wandering was positively related to MVPA (β=3.74, SE=0.23; *t*_1842.8_=15.96, *P*<.001), whereas this relationship was negative in nature during times of negative affect (β=–3.02, SE=0.23, *t*_1842.8_=–12.87, *P*<.001; see Figure 2). The residual pseudo *R*^2^ indicated that this model accounted for a moderate amount of within-person variance (pseudo *R*^2^=.06 [39]).

**Figure 3:**
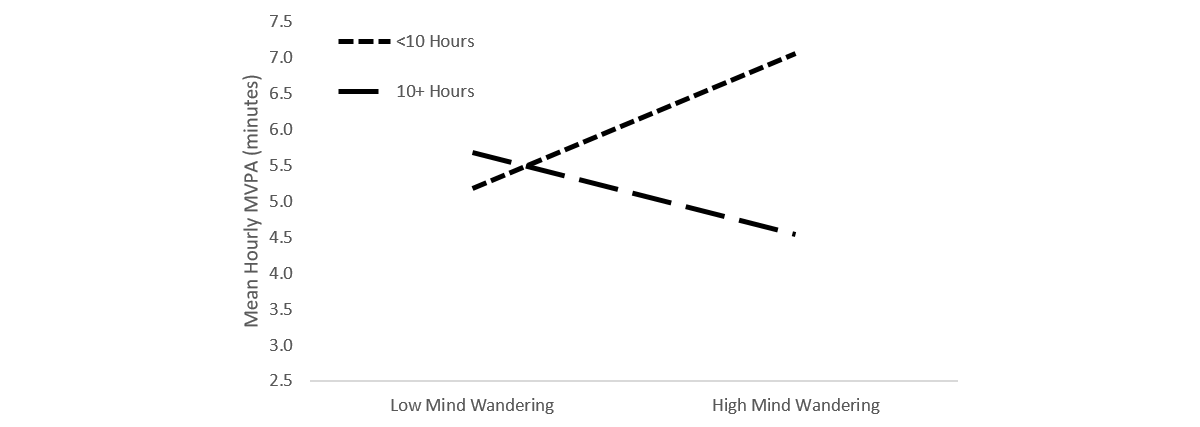
Interaction between mind wandering and sleep duration.

Although daily sleep quality was unrelated to MVPA (*P*=.17), there was a negative association between nightly sleep duration and MVPA during the following day (*P*=.001), a finding that is in line with numerous epidemiological studies relating excessive sleep to lower physical activity participation rates [40-42]. To further investigate this finding, we examined nights with overly long sleep duration (ie, <10 hours per night=1 vs ≥10 hours per night=–1 [42,43]; see model 2). This “oversleep” was associated with less physical activity on the following day (*P=*.007). The interaction between long sleep duration and mind wandering was not statistically significant (*P=*.07), but further investigation via simple slopes analyses indicated that for those who overslept, mind wandering was unrelated to physical activity (β=–0.31, SE=0.40; *t*_1797.8_=0.76, *P=*.45). Among those who did not oversleep, mind wandering was significantly and positively related to physical activity (β=0.50, SE=0.19; *t*_1797.8_=2.69, *P=*.007; see Figure 3). These relationships were not present when alternative sleep duration cutoffs were applied (eg, ≥9 hours; *P*=.27). No demographic items were associated with MVPA (all *P* s ≥.11). The final model, when compared with the unconditional means model, accounted for a small-to-moderate amount of within-person variance (pseudo *R*^2^=.05 [39]). The model fit, fixed effects, and random effects for the unconditional means model and important model steps are displayed in Tables 2-4.

**Table 2:** Model fit statistics for mixed models associated with moderate-to-vigorous physical activity.

Model fit	Unconditional means model	Model 1	Model 2
–2LL	17,216.30	11,846.30	11,655.82
AIC	17,222.30	11,868.30	11,681.82
BIC	17,239.99	11,929.14	11,753.51
Pseudo *R*^2^ within	NA	.08	.05

**Table 3:** Fixed effects for mixed models associated with moderate-to-vigorous physical activity.

Fixed effects		Unconditional means model			Model 1			Model 2		
		β (SE)	*t* (*df*)	*P*	β (SE)	*t* (*df*)	*P*	β (SE)	*t* (*df*)	*P*
**Within-person**										
	Intercept	4.72 (0.25)	19.09 (32.85)	<.001	5.95 (0.51)	11.74 (51.95)	<.001	5.62 (0.53)	10.68 (58.07)	<.001
	Linear time				–0.18 (0.05)	–3.90 (15.18)	.001	–0.17 (0.05)	–3.59 (27.96)	.001
	Quadratic time				–0.04 (0.01)	–3.97 (1836.18)	<.001	–0.04 (0.01)	–4.08 (1805.99)	<.001
	Day of the week				0.17 (0.07)	2.58 (1830.15)	.01	0.17 (0.07)	2.42 (1803.66)	.02
	Hourly mind wandering				0.36 (0.17)	2.15 (1831.54)	.03	0.10 (0.22)	0.44 (1798.93)	.66
	Hourly feelings				0.19 (0.08)	2.26 (1823.58)	.02	0.21 (0.09)	2.45 (1802.09)	.02
	Feeling*mind wandering				0.21 (0.10)	2.07 (1842.83)	.04	0.23 (0.10)	2.19 (1815.89)	.03
	Oversleep							0.51 (0.19)	2.72 (1412.30)	.007
	Mind wandering*oversleep							0.41 (0.22)	1.82 (1797.80)	.07

**Table 4:** Random effects for mixed models associated with moderate-to-vigorous physical activity.

Random effects	Unconditional means model			Model 1			Model 2		
	β (SE)	Wald *Z*	*P*	β (SE)	Wald *Z*	*P*	β (SE)	Wald *Z*	*P*
**Residual**	34.58 (0.95)	36.45	<.001	32.55 (1.09)	29.95	<.001	32.69 (1.10)	29.68	<.001
Intercept	1.59 (0.50)	3.19	<.001	3.86 (1.61)	2.40	.02	3.94 (1.64)	2.40	.02
Linear time				0.03 (0.02)	1.45	.15	0.03 (0.02)	1.46	.14
Rho				–0.79 (0.13)	–5.91	<.001	–0.82 (0.12)	–6.96	<.001

## Discussion

### Principal Findings

This study is the first to examine the associations between mind wandering, sleep, affect regulation, and MVPA. The results offer preliminary support for our primary hypothesis. Specifically, they suggest that daily mind wandering in conjunction with positive affect is positively associated with MVPA among college students. These early and exploratory findings appear to add support to previous laboratory-based research that highlighted the often-prospective nature of mind wandering, which supports planning of personally relevant future goals [2], by demonstrating associations between mind wandering and a complex and important self-regulated behavior in the real world. That the impact of mind wandering on physical activity is influenced by the individual’s affective state and sleep is a novel finding that may have implications for health researchers.

These findings provide support for relatively recent recommendations made by Smallwood and Andrews-Hanna [6]. The authors advise researchers to take a nuanced view of mind wandering. Clearly, the ability to disengage from the present is required for thinking about and planning for the future, which is a necessary daily behavior when carrying out challenging and complex behaviors such as physical activity [18]. Still, when an individual finds they “feel” badly, have slept poorly, or are in a cognitively demanding situation, these thoughts are likely to move away from such beneficial, future-directed thought [2,44]. By recognizing these important determinants, interventionists can begin to develop strategies to nudge individuals away from unproductive mind wandering, perhaps impacting a number of outcomes such as program adherence, long-term behavioral maintenance, or academic performance.

Better sleep quality was unrelated to physical activity, which was counter to our initial hypothesis given the relationships between sleep quality and physical activity, as well as with a number of related constructs. For instance, sleep has been associated with a number of aspects of cognition believed to be necessary for self-regulation (eg, working memory, problem solving, attention-set shifting) [20,21]. The finding related to excess sleep duration, however, was less surprising. Poor sleep hygiene is common among college students [28], and is marked by irregular wake and sleep times as well as too little or too much sleep [45]. Numerous studies have found overly long sleep to be related to a number of negative outcomes, including greater risk for heart disease [42], less physical activity [40,46], depression, and overall morbidity and mortality [41]. In this study, students tended to engage in less MVPA on days following sleep lasting 10 or more hours. Additionally, the interaction between extended sleep and mind wandering approached significance, and indicated that mind wandering was positively related to physical activity only among those that slept less than 10 hours per night.

### Implications for Future Work

These findings lay the groundwork for future research examining the efficacy of a multiple health behavior change approach to improving physical activity and sleep habits concurrently. Over the last decade, a number of efficacious and scientifically sound Web-based sleep therapy tools have been developed (eg, SHUTi [47]), and it would be quite feasible to integrate such approaches with a physical activity intervention. Additionally, these findings hint at the possible reciprocal nature of the relationships between mind wandering, affect regulation, sleep, and physical activity. Although exercise is a common method for improving sleep hygiene [28,48-52], improved sleep habits may support prospective mind wandering, in turn promoting physical activity behavior and ultimately contributing to better sleep. Although sleep quality was unrelated to physical activity and mind wandering in this study, it was assessed via daily self-report. Future research utilizing objective measures of sleep and sleep quality may shed important light on these relationships.

Although it was outside of the scope of this study, working memory capacity and its relation with mind wandering and physical activity is another promising target for future research as several researchers have found this construct to be related to affect and self-regulation [53-55]. Carpenter et al [55] manipulated the affective state of 46 older adults, reporting that those in the positive-feeling condition performed better on tasks of complex decision making and working memory capacity relative to individuals in a neutral-control condition. These working memory skills are necessary for the maintenance of goals over time, and researchers have demonstrated those with greater working memory capacity can not only limit or inhibit intrusive thought, but may also better downregulate the effects of “hot” processes such as negative affect and cravings [18,56]. These individuals are often more capable of disengaging from information that may distract them from self-regulatory efforts [57].

Mindfulness training may offer one promising strategy for influencing affect, bolstering working memory, and biasing mind wandering toward prospective thought [58-61]. Importantly, these strategies might also be readily introduced into a physical activity intervention. Although definitions vary, the mental state of mindfulness is often characterized by the devotion of full attention to the present without judgment or emotional reactivity [58]. Jha and colleagues [58] administered 8 weeks of mindfulness training to members of the military readying for deployment. The researchers reported that greater time spent practicing mindfulness meditation was related to lower levels of negative affect and with increased working memory capacity, and the association between affect and mindfulness training was mediated by working memory capacity. If successful, the application of such training methods in the context of a physical activity intervention has potential to increase the likelihood of future-oriented mind wandering, perhaps improving study outcomes. The provision of these self-regulation supporting skills may also improve long-term maintenance of the behavior, which is a recurring challenge for health behavior researchers [62].

### Strengths and Limitations

We believe this study has a number of strengths. It is the first to establish relationships between mind wandering, affect regulation, sleep, and physical activity. Additionally, physical activity was measured objectively, and the use of mobile phones allowed for the examination of these relations as individuals moved throughout their daily lives over the course of 1 week. Finally, the population of college students recruited for this study was quite diverse, with approximately one-half being nonwhite.

We do acknowledge several limitations present in this exploratory study. First, although many assessments were collected for each participant, the overall sample size was rather small, potentially impacting our ability to draw conclusions related to between-persons relationships. Similarly, due to the large number of daily physical activity assessments, participants may have experienced some degree of measurement reactivity [63], in turn temporarily altering their behavior in response to the measurement. To ensure the effects of this study were not impact by reactivity, future researchers may consider the inclusion of supplementary assessment groups (eg, EMA only, activity monitor only). Additionally, the sample was college-aged and largely female (75%), potentially reducing the broader generalizability of these findings; further work would benefit by extending this assessment to nonstudent populations. The decision to use a high rate of prompting may have contributed to rather high degree of missing data. Future researchers may consider the use of longer study periods with more contemporary prompting schemes (eg, random prompting, prompting based on individual schedules) to reduce missingness. Further, in accordance with our primary aims, we collected assessments of self-reported mind wandering, but we did not collect assessments of the content of an individual’s thoughts. Thus, we are unable to draw clear ties between the specific nature of mind wandering and future behavior. Doing so may add further richness to our understanding of the nature of the relationships demonstrated in this study. Similarly, as was previously noted, accelerometers were worn during waking hours only. As a result, only daily self-reported measures of sleep were used in analyses. By utilizing more objective measures of sleep, researchers may better understand the relationships between sleep, mind wandering, and physical activity. It is also likely that working memory plays an important role in the relations between mind wandering and physical activity. With the growing popularity of mobile cognitive assessments, it is becoming increasingly feasible to collect multiple assessments of working memory over time, potentially shedding light on the temporal relations between working memory, mind wandering, and health behaviors such as physical activity.

Finally, this exploratory work was concerned specifically with the relationships between mind wandering and physical activity behavior. However, it is important to note this important health behavior does not occur in a vacuum. Indeed, it is likely to operate synergistically with a number of other health behaviors, including sleep, dietary behaviors, substance use behaviors, and social activities [64]. One’s daily activity behaviors may also be affected by myriad psychosocial factors (eg, depression, anxiety, loneliness). The interesting relationships highlighted in this analysis underscore the novelty of this area of research and lay the initial groundwork for additional research into the influences of these important factors.

### Conclusions

Our findings suggest the degree to which an individual’s mind wanders is associated with their participation in MVPA and this relationship is influenced by both affect and sleep duration. Future physical activity research implementing strategies to improve attention, affect regulation, and sleep hygiene is warranted, potentially offering important information for those attempting to begin or maintain a physically active lifestyle.

## Abbreviations

AIC: Akaike information criterion
BIC: Bayesian information criterion
EMA: ecological momentary assessment
MVPA: moderate-to-vigorous physical activity
